# Efficacy and safety of sodium-glucose cotransporter-2 inhibitors in type 2 diabetes mellitus with inadequate glycemic control on metformin: a meta-analysis

**DOI:** 10.20945/2359-3997000000146

**Published:** 2019-06-19

**Authors:** Zhang Jingfan, Li Ling, Liu Cong, Li Ping, Chen Yu

**Affiliations:** 1 Department of Endocrinology Shengjing Hospital China Medical University Shenyang Liaoning Province China Department of Endocrinology, Shengjing Hospital of China Medical University, Shenyang, Liaoning Province, China

**Keywords:** Sodium-glucose co-transporter 2 inhibitors, metformin, type 2 diabetes mellitus, meta-analysis

## Abstract

**Objectives:**

To provide a meta-analysis of the clinical efficacy and safety of sodium glucose co-transporter 2 inhibitors (SGLT2-i), as a combination treatment with metformin in type 2 diabetes mellitus (T2DM) patients with inadequate glycemic control with metformin alone.

**Materials and methods:**

We have searched randomized controlled trials (RCTs) in the database: MEDLINE, Embase and Cochrane Collaborative database. We used mean differences (MD) to assess the efficacy of glycemic and other clinical parameters, and risk ratios (RR) to evaluate the adverse events for safety endpoints. The heterogeneity was evaluated by I^2^.

**Results:**

Finally 9 studies were included. SGLT2-i plus metformin had higher reduction level in HbA1C [MD = -0.50, 95% CI (-0.62, -0.38), p < 0.00001], FPG [MD = -1.12, 95%CI (-1.38, -0.87), p < 0.00001], body weight [MD = -1.72, 95% CI (-2.05, -1.39), p < 0.00001], SBP [MD = -4.44, 95% CI (-5.45, -3.43), p < 0.00001] and DBP [MD = -1.74, 95% CI (-2.40, -1.07), p < 0.00001] compared with metformin monotherapy. However, SGLT2-i plus metformin group had higher risk of genital infection [RR = 3.98, 95% CI (2.38, 6.67), p < 0.00001]. No significant difference was found in the risk of hypoglycemia, urinary tract infection or volume related adverse events.

**Conclusions:**

Although the risk of genital infection may increase, SGLT2-i plus metformin may provide an attractive treatment option to those T2DM patients who are unable to achieve glycemic control with metformin alone, based on its effects on glycemic control, reducing body weight and lowering blood pressure.

## INTRODUCTION

Type 2 diabetes mellitus (T2DM) is a kind of chronic and progressive disease with high prevalence. With the rapid economic development and urbanization, T2DM is on the rise all over the world. International Diabetes Federation (IDF) has estimated that, some 425 million (8.8%) adults, 20-79 years old, were likely to have diabetes worldwide. There will be 629 million diabetic patients by 2045. About 87% to 91% diabetic patients are probably T2DM in high income countries (1).

Simplistically, in T2DM, hyperglycemia is caused by the following two reasons: inadequate insulin secretion and insulin resistance (the body cannot fully respond to insulin). If attempts to change lifestyle, the cornerstone of T2DM treatment, are not able to control levels of blood glucose, metformin will be usually treated as the most commonly initial oral medication worldwide (1). Nevertheless, there are still 33–49% diabetic patients failed to meet glycemic target, as well as blood pressure control or cholesterol target (2).

Sodium glucose co-transporter 2 inhibitors (SGLT2-i), as a novel kind of antidiabetic drugs, provide a new way to treat those T2DM patients unmet control target. SGLT2-i was recommended as second-line agents in T2DM management by American Diabetes Association (ADA) and European Association for the Study of Diabetes (EASD) in the year of 2015 (3). The antidiabetic effect of SGLT2-i is based on inhibiting glucose reabsorption in renal proximal tubules, increasing the excretion of urinary glucose, thus reducing the blood glucose (4). Several human trials demonstrated that SGLT2-i could decrease the blood glucose levels and glycated hemoglobin (HbA1C) levels irrespective of the amount or sensitivity of insulin, consequently improved the glycemic control (5,6). As a result, all other kinds of antidiabetic agents can be combined with SGLT2-i, which include exogenous insulin in them (5,6). During the traditional therapy for T2DM, the side effects, such as hypoglycemia and weight gain, frequently occurred. Therefore, the benefits of glycemic control by the treatments may be negated. Nevertheless, SGLT2-i can improve the control of blood glucose without causing the above side effects because that the factors of hypertension, glomerular hyperfiltration and weight gain are controlled by SGLT2 gene (6). Up to now, the following SGLT2-i have been approved in one major market (such as the European Union, the United States, and Japan) at least, including canagliﬂozin, dapagliﬂozin, empagliﬂozin, luseogliﬂozin, ipragliﬂozin, and tofogliﬂozin (7-9). However, large trials evaluating the efficacy and safety of SGLT2-i combined with metformin are still lacking. We conducted the present meta-analysis to update and synthesize the efficacy and safety of SGLT2-i, as add-on to metformin in T2DM patients with inadequate glycemic control in metformin alone.

## MATERIALS AND METHODS

We have searched randomized controlled trials (RCTs) in the database: MEDLINE (1978 to November 2017), Embase (1974 to November 2017) and Cochrane Collaborative database, based on the following search terms: ‘sodium glucose co-transporter 2 inhibitors’, ‘SGLT2 inhibitors’, the names of individual available SGLT2-i (‘canagliﬂozin’, ‘CANA’, ‘dapagliﬂozin’, ‘DAPA’, ‘empagliﬂozin’, ‘EMPA’, ‘luseogliﬂozin’, ‘ipragliﬂozin’, ‘IPRA’ and ‘tofogliﬂozin’, ‘TOFO’) and ‘metformin’.

The studies met the following criteria were included: [1] RCTs recruited adult patients of T2DM with inadequate glycemic control on metformin, [2] Studies compared SGLT2-i as add-on to metformin with placebo combined with metformin, [3] Treatment duration ≥ 12 weeks, [4] The following data was completely reported: the change of fasting plasma glucose (FPG), the change of HbA1C, the change of body weight, the change of systolic blood pressure (SBP) and diastolic blood pressure (DBP), special interest adverse events (AEs) of SGLT2-i included hypoglycemia AEs, AEs suggestive of urinary tract infection (UTI), AEs suggestive of genital infection (GI), and volume related AEs-hypotension/dehydration/ hypovolemia.

In order to evaluate the relevance, two independent investigators reviewed abstracts of articles. If judged pertinent, articles were further taken into account. We tried to identify and resolve the discrepancies or disagreements by discussion and consensus. If needed, a third investigator would participate in the discussion and confirm by consensus. If multiple articles were attached to the same trial, we chose the most recently published or most complete data. We used Revised Jadad’s Scale to assess the quality of included articles, with scores range from 0 to 7 (a high score indicating high quality).

We used Review Manger (version 5.3; Cochrane collaboration) to perform all statistical analyses. For the measurement of efficacy, we calculated the weighted mean differences (MD) and 95% confidence interval (CI) of the mean changes from baseline of the following continuous variables: FPG, HbA1C, body weight, SBP and DBP. For the measurement of safety, we used risk ratio (RR) with 95% CI to assess the dichotomous variables (hypoglycemia, AEs of UTI, AEs of GI, and volume related AEs-hypotension/ dehydration/ hypovolemia). We performed Q-statistic test (significant level at p < 0.05) and I^2^ tests (I^2^ ≥ 50% reveal a substantial level of heterogeneity) to evaluate the heterogeneity among trials (10). We performed subgroup analyses of different individual SGLT2-i agents to evaluate the confounding effect of heterogeneity. We used Random-effects model in the assessment of continuous variables of HbA1C, FPG and body weight, since statistical heterogeneity presented in the analyses. Fixed-effects model was used in the analyses of continuous variables of SBP and DBP, and dichotomous variables with little heterogeneity.

## RESULTS

**Figure 1:**
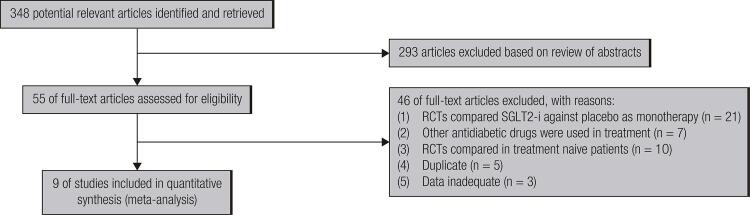
Flow diagram of study selection process.

We identified 348 RCT citations initially, after review of abstracts, 293 articles were excluded, 55 articles were further assessed for evaluation in detail based on review of the full text. However, 46 articles were excluded for the following reasons: [1] RCTs comparing SGLT2-i against placebo controlled group as monotherapy; [2] -Besides SGLT2-i or metformin, other antidiabetic drugs were used in treatment; [3] RCTs comparing SGLT2-i with metformin in treatment naive patients; [4] Duplicate; [5] The data about outcomes of efficacy and safety was inadequate. Finally, 9 studies (11-19) with total 2509 patients were included for meta-analysis (Figure 1). None of luseogliﬂozin or tofogliﬂozin were finally included in the meta-analysis since no studies met the inclusion criteria. The mean revised Jadad’s score of 9 included RCTs was 5.4, and seven of nine studies had a score ≥ 5, which demonstrated the adequate methodologic quality of the enrolled studies. The basic characteristics of patients enrolled, and information on drug therapy of included studies were presented in Table 1.

**Table 1:** Basic characteristics of included studies

Study (year)	Age (years) Mean ± SD			Male (%)		Study duration (weeks)	Number of patients		Dosage		Revised Jadad score
	SGLT2-i plus MET	PBO plus MET		SGLT2-i plus MET	PBO plus MET		SGLT2-i plus MET	PBO plus MET	SGLT2-i	MET	
Lu CH (11) (2016)	53.9 ± 11.3	53.4 ± 11.3		50.6	39.8	24	87	83	IPRA 50 mg/d	≥ 1500 mg/d (or ≥ 1000 mg/d if safety concerns prohibited higher doses)	7
Bailey CJ (12) (2013)	54.4 ± 9.4	53.7 ± 10.3		50	55	102	137	137	DAPA 5 mg/d	≥ 1500 mg/d	7
Lavalle-González FJ (13) (2013)	55.5 ± 9.4	55.3 ± 9.8		47.3	51.4	26	368	183	CANA 100 mg/d	≥ 2000 mg/d (or ≥ 1500 mg/d if unable to tolerate higher dose)	6
Wilding JP (14) (2012)	58.6 ± 7.6	57.3 ± 8.6		47.1	54.5	12	68	66	IPRA 50 mg/d	≥ 1500 mg/d	3
Rosenstock J (15) (2013)	59 ± 9.0	60 ± 8.5		47	47	12	71	71	EMPA 10 mg/d	≥ 1500 mg/d or maximum tolerated dose	5
Merker L (16) (2015)	55.5 ± 9.9	56 ± 9.7		57.6	56	76	217	207	EMPA 10 mg/d	≥ 1500 mg/d or maximum dose according to the local label	5
Schumm-Draeger PM (17) (2015)	58.3 ± 9.0	58.5 ± 9.4		37	46.5	16	100	101	DAPA 5 mg/d	≥ 1500 mg/d	7
Ross S (18) (2015)	58.5 ± 10.8	57.9 ± 11.2		50.5	51.4	16	214	107	EMPA 10 mg/d	≥ 1500 mg/d	3
Yang W (19) (2016)	53.1 ± 9.1	53.5 ± 9.2		45.6	59.3	24	147	145	DAPA 5 mg/d	≥ 1500 mg/d	6

SGLT2-i: sodium-glucose co-transporter 2 inhibitors; MET: metformin; PBO: placebo; IPRA: ipragliﬂozin; DAPA: dapagliﬂozin; CANA: canagliﬂozin; EMPA: empagliﬂozin.

**Figure 2:**
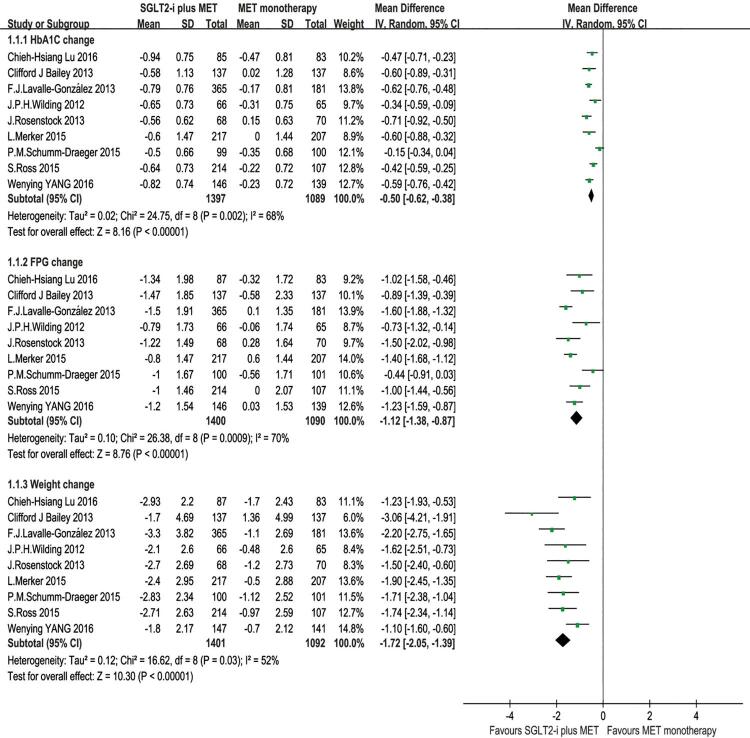
Meta-analysis of efficacy in changing HbA1C, FPG and weight from baseline.

**Figure 3:**
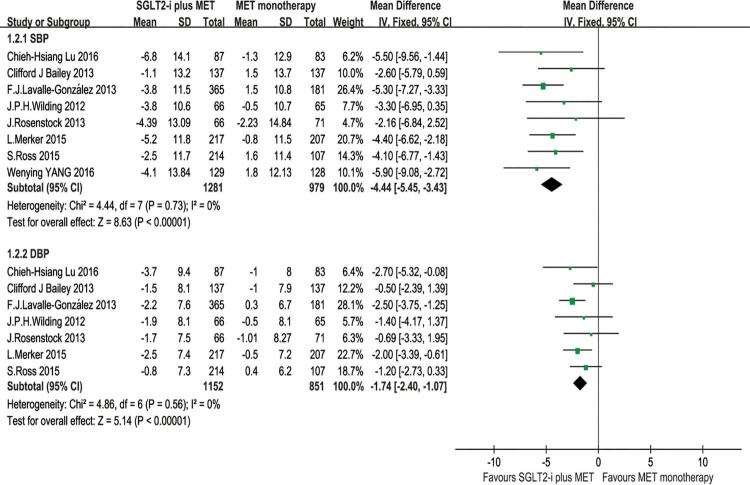
Meta-analysis of efficacy in changing SBP and DBP from baseline.

For the comparison of efficacy between SGLT2-i plus metformin and metformin monotherapy as treatment in T2DM patients with inadequate glycemic control on metformin alone, we analyzed the changes from baseline of HbA1C, FPG and body weight, and all of the 9 articles were enrolled in the analysis. The results were shown in Figure 2. The efficacy results of our meta-analysis showed that the SGLT2-i combined with metformin had higher reduction level in HbA1C (%) [MD = -0.50, 95% CI (-0.62, -0.38), p 0.00001], however, with a great quantity of heterogeneity (I^2^ = 68%); higher reduction in FPG level (mmol/L) [MD = -1.12, 95% CI (-1.38, -0.87), p < 0.00001], but with a great quantity of heterogeneity (I^2^ = 70%); higher reduction in body weight (kg) [MD = -1.72, 95% CI (-2.05, -1.39), p < 0.00001], with heterogeneity (I^2^ = 52%) compared with metformin monotherapy (Figure 2). We also compared the changes from baseline of SBP and DBP, and found that SGLT2-i plus metformin got higher reduction in SBP [MD = -4.44, 95% CI (-5.45, -3.43), p < .00001] and DBP [MD = -1.74, 95% CI (-2.40, -1.07), P < 0.00001] compared with metformin monotherapy (Figure 3).

**Figure 4:**
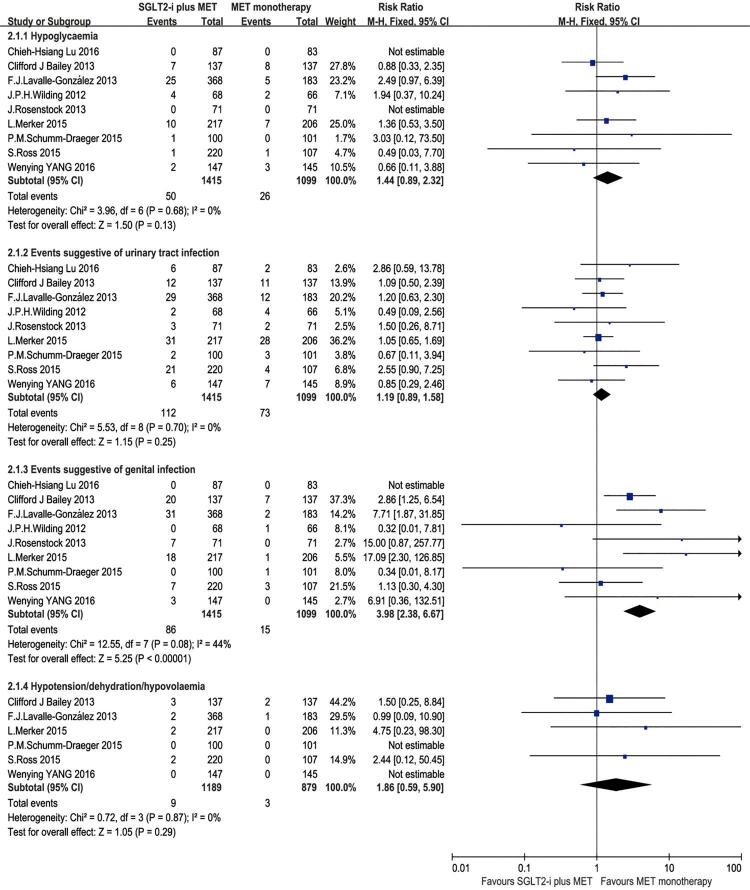
Meta-analysis of of safety in the incidence of special interest adverse events.

For the comparison of safety, we analyzed the risk ratios of special interest AEs of SGLT2-i, included hypoglycemia, AEs of UTI, AEs of GI, and volume related AEs-hypotension, dehydration or hypovolemia, shown in Figure 4. The meta-analysis results showed no significant difference between the SGLT2-i plus metformin group and the metformin monotherapy in the incidence risk of hypoglycemia [RR = 1.44, 95%CI (0.89, 2.32), p = 0.13], or the risk of AEs of UTI [RR = 1.19, 95% CI (0.89, 1.58), p = 0.25], nor the risk of volume related AEs [RR = 1.86, 95% CI (0.59, 5.90), p = 0.29]. However, SGLT2-i plus metformin group presented higher risk of AEs of GI [RR = 3.98, 95% CI (2.38, 6.67), p < 0.00001], compared with the group of placebo plus metformin (Figure 4).

## DISCUSSION

We performed this meta-analysis to examine the efficacy and safety of SGLT2-i as combination therapy with metformin in those T2DM patients with inadequate glycemic control on metformin alone. The results showed a significant effect of SGLT2-i plus metformin as combination treatment in improving T2DM patients’ HbA1C, FPG, body weight and blood pressure. However, SGLT2-i plus metformin group showed higher risk of genital infection. No significant difference in the risk of hypoglycemia, UTI or the risk of volume related AEs was found. In summary, the combination therapy of SGLT2-i and metformin presented better efficacy than metformin monotherapy, although with a higher risk of genital infection.

Metformin is recommended as the first line drug for T2DM patients with insufficient glycemic control after lifestyle interventions. However, due to the T2DM progression, it may not provide adequate glycemic control which necessitates add-on treatments. According to the ADA and the American Association of Clinical Endocrinologists (AACE) guidelines, metformin may be followed by sulfonylurea, GLP-1 receptor agonists, SGLT2-i, DPP-4 inhibitors, thiazolidinediones, or basal insulin (20,21). Among the recently developed drugs, SGLT2-i have shown promising results for T2DM patients (22-24). Selective and reversible inhibition of SGLT2 can lower blood glucose levels independent of insulin status and is also found to manifest favorable effects on hypertension and body weight control, besides maintaining glycemic control. Several RCTs have found the effect of SGLT2-i on decreasing HbA1C, FPG, and body weight by inducing favorable glucosuria (urinary loss of approximately 200–300 kcal/d), compared with placebo or other glucose-lowering drugs (25-30). In the present study, we only analyzed the data of SGLT2-i with recommended minimum daily doses (IPRA 50 mg/d, DAPA 5 mg/d, CANA 100 mg/d, EMPA 10 mg/d), because we regarded these dosages as the most widely used as initial treatment of SGLT2-i in clinical work. Higher doses might overestimate the effectiveness or the risk of adverse effect of SGLT2-i. As shown in the results, SGLT2-i even in low doses, when combined with metformin, showed a significant reduction of HbA1C, FPG, body weight and blood pressure. These results suggested that combination treatment of SGLT2-i and metformin can provide benefits to patients having inadequate control on T2DM with metformin, especially those with hypertension and/or obesity.

Despite the glycemic control, we have noted a significantly higher incidence of genital infections in SGLT2-i plus metformin group compared with metformin monotherapy. The slightly higher incidence of urogenital infections in SGLT2-i treated T2DM has been reported after analysis of pooled data from phase III trials (31). It was thought to be due to increased urinary glucose which may act as a potential fungal growth factor in SGLT2-i treated patients (32). These studies have raised concerns about the safety testing of SGLT2-i with regards to the higher incidence of genital infections (33,34). We didn’t find the higher risk of urinary tract infection in SGLT2-i plus metformin compared with metformin monotherapy. However, we should fully evaluate the benefits and harms of SGLT2-i when provide it to patient therapy, especially those women patients with a history of chronic or recurrent genital infection. What’s more, counseling patients about genital hygiene is likely to minimize the risk of infection.

Some further concerns were generated by reports of ketoacidosis associated with SGLT2-i (35,36). SGLT2-i stimulate the release of glucagon, thus increasing the production of ketone bodies (37). Furthermore, the inhibition of SGLT-2 stimulates ketone re-absorption in the renal tubule (38). As a result, treatment with SGLT2-i could be associated with increased ketonemia, leading to acidosis. Cases of ketosis without severe hyperglycemia have been reported during treatment with SGLT-2 inhibitors (35), inducing regulatory authorities to issue a warning (36). However, no related information was reported in our included articles, we didn’t assess the risk of ketoacidosis. Furthermore, the possible effects of SGLT2-i on the renal tubular transportation of bone minerals are also being concerned. Only two of our included articles reported the AEs of fractures (17,19). Schumm-Draeger PM reported no fracture accident in the study (17). Yang reported one fracture in MET monotherapy with total 145 patients in the group, while no fracture reported in DAPA 5 mg/d plus MET group with total 147 patients (19).

There are limitations in our present study. We collected the data of meta-analysis based on the published articles in journal. It might introduce the publication bias as the ‘positive’ findings were more likely to be reported. However, RCTs may have relatively low risk of such kind of bias. Another constraint is the lack of some outcomes from the enrolled studies, particularly those outcomes we were interested in, such as the renal effects of SGLT2-i treatment, the incidence of cardiovascular events, ketoacidosis, and so on. Moreover, another concern is related to statistical heterogeneity (I^2^) which was relatively high in the comparison of the change in HbA1C (I^2^ = 68%), FPG (I^2^ = 70%) and body weight (I^2^ = 52%). While in the other analyses, it was either absent or low. This heterogeneity can be attributed to clinical and methodological heterogeneity. Across different RCTs, there could be differences in some extent in the ethnicities, treatment duration, and/or differences in the selection and ascertainment of outcomes. Furthermore, the trials were multiregional, and each center contributed a relatively small number of patients, which might lead to a high variability in the overall analysis. Future ideal RCTs are necessary, which should compare SGLT2-i with placebo or other diabetic therapies in longer time and larger scale, with adequate follow-up, and report all the related data including renal safety, cardiovascular events, ketoacidosis and other related adverse events, so as to evaluate the long-term consequences of SGLT2-i treatment.

Overall, the present meta-analysis evaluated the effectiveness and safety of SGLT2-i plus metformin treatment, compared with metformin monotherapy in the T2DM patients with inadequate glycemic control on metformin alone. In conclusion, although the risk of genital infection may increase, SGLT2-i plus metformin may provide an attractive treatment option to those T2DM patients who are unable to achieve glycemic control with metformin alone, on account of its effects on glycemic control, reducing body weight and lowering blood pressure. Further researches are necessary to clarify the long-term efficacy of this biotherapy and the potential risk of the therapeutic intervention.
